# A Peculiar Case

**Published:** 1864-01

**Authors:** A. S. Talbert


### A PECULIAR CASE.

By A. S. Talbert, D. D. S.

August 12, 1863.—Filled the proximal surfaces of the right superior central and lateral incisors, with *great care*, for Miss E., æt. about 19 years—of whose teeth I have long had the charge.

She had previously separated them with elastic gum, which, with the little filing necessary to remove the softened, fringed edges of the enamel, gave me good room to operate.

No unusual sensibility was observed in either tooth, though past experience had taught me to expect the pulp larger than in many persons of the same age. Neither saw nor heard anything from patient till the 6th instant, when she called with a younger sister. While in my office, I embraced the occasion to examine those teeth last filled; when, to my great surprise, I found in the lateral incisor a *black* line running nearly the entire circle of the filling, and between it and the edges of the enamel! Requested patient to again separate them with the elastic gum. Returned five days after; removed the filling, *en masse*, and found the *whole* surface of the cavity covered with a black pigment, very thin but black as any oxide, granulated and resembling the tartar that adheres so tenaciously to artificial teeth and plates—(I wish somebody would tell me how to get it off)—after heating them to mend, after they have been worn and broken.

Removed this lining with instrument and thorough washing. The dentine apparently about as sensitive as when the tooth was filled five months ago! Two little black specks remained at points in the cavity nearest the pulp, barely large enough to be seen with the naked eye. These were allowed to remain, lest, in their removal, I might actually expose the pulp. The tooth was again filled.

Whence came this deposit? and what is it? Do these spots indicate minute openings which were invisible to the eye till blackened by a vitiated serum ejected from the pulp —a result of an inappreciable wound to its investing membrane? There has been no pain or apparent inflammation. The vitality is neither destroyed nor apparently impaired. This colored substance did not come from oxidation of the gold. It did not adhere to the filling, but to the bottom and walls of the cavity. How did it get there? Can not I *fill a tooth* so that even serum will not penetrate between the filling and the entire walls of the cavity?—(Will somebody be kind enough to tell me *how to fill a tooth?*) But if I had *perfectly filled it*, what would have become of this coloring matter? I remark, in conclusion, the filling in the *central* incisor *seems* to have been well done.

